# Backyard poultry cases in UK small animal practices: Demographics, health conditions and pharmaceutical prescriptions

**DOI:** 10.1002/vetr.71

**Published:** 2021-01-28

**Authors:** David A. Singleton, Christopher Ball, Cameron Rennie, Charlotte Coxon, Kannan Ganapathy, Phil H. Jones, David Welchman, John S.P. Tulloch

**Affiliations:** ^1^ Infection, Veterinary and Ecological Sciences University of Liverpool Cheshire UK; ^2^ International Disease Monitoring and Risk Assessment (EU Exit) Animal and Plant Health Agency Addlestone UK; ^3^ Surveillance Intelligence Unit Animal and Plant Health Agency Addlestone UK; ^4^ Surveillance Intelligence Unit Animal and Plant Health Agency Winchester UK

## Abstract

**Background**: Backyard poultry ownership is of keen interest in the United Kingdom. However, despite this, little is known about veterinary care engagement and outcomes of visits in this group of species.

**Methods**: This study described and characterised veterinary practice‐visiting backyard poultry, utilising electronic health record data supplied by veterinary practices voluntarily participating in the Small Animal Veterinary Surveillance Network between 1st April 2014 and 31st March 2019. **Results**: In total, 4424 recorded poultry consultations originating from 197 veterinary practices (352 sites) were summarised. Chicken consultation (n = 3740) peak incidence was in early summer (April‐June), relative to all recorded species. More chickens resided in rural (incident rate ratio = 2.5, confidence interval [CI] 2.3–2.6, *p* <0.001) or less deprived areas. Non‐specific clinical signs were commonly recorded (17.6% of chicken consultations, CI 15.9–19.2), as were those indicative of advanced disease. This latter finding was reflected in prescribed management strategies, with euthanasia comprising 29.8% (CI 27.0–32.6) of consultations. Antimicrobials were commonly prescribed (33.0% of consultations, CI 29.8–36.2), 43.8% of which included antimicrobials considered ‘highest priority critically important’ by the World Health Organisation.

**Conclusion**: Our findings indicate a need to tailor antimicrobial prescription guidance to the backyard poultry setting. In addition, late presentation of disease, vague clinical descriptions in clinical narratives and high euthanasia rates show that disease identification, management and knowledge of poultry health and welfare among owners and veterinary surgeons can be improved.

## INTRODUCTION

Domestic fowl ownership accounts for the fourth highest pet population in the United Kingdom, with an estimated 0.4% of households owning domestic fowl in 2019.^1^ Such birds include purchased ex‐laying hens and exotic breeds. Despite the popularity of domestic fowl, clinical and demographic information on the nationwide backyard population is lacking. According to governmental legislation, only flock sizes of 50 or over are required to be registered with the Animal and Plant Health Agency (APHA).^2^ Between 2011 and 2013, a total of 37,086 premises were registered in Great Britain, with 17,259 premises voluntarily registered as having less than fifty birds.^3^ An estimated backyard population of 3 million birds has also been quoted,^4^ although as registration is voluntary, this sub‐population of poultry ownership may be underrepresented.^5^

Poultry pathogens can be transmitted between backyard flocks through several routes, mostly through the purchase and sale of birds, movement of birds through markets, presentation at shows and wild birds.^6^, ^7^ For example, a study of chicken fanciers in Belgium identified a high percentage of small chicken flocks were positive for respiratory pathogens by both antibody and antigen detection.^8^ Inadequate biosecurity may also increase disease transmission risk.^9^, ^10^ Recent work has shown that viruses, such as infectious bronchitis virus (IBV) and avian metapneumovirus, and bacterial pathogens, such as mycoplasmas, are present in UK commercial farms,^11^ and many of the same respiratory pathogens can also be found in backyard and small chicken flocks.^12^ In the UK, backyard owners tend to source their poultry from various places, including ‘rehoming’ commercial hens. Early clinical signs resulting from such infections can be non‐specific, especially for respiratory or reproductive tract infections.^4^ Such signs may potentially go unnoticed by owners,^13^ unless they cause a substantial reduction in egg production. Owners of backyard chickens may not have access to specialist poultry veterinarians, and the vet‐visiting population is more likely to consult a nearby companion animal veterinary surgeon as a result.^14^ Alternatively, owners may also rely on advice from the internet or social media.^13^, ^14^, ^15^

Similar to other agricultural sectors, commercial poultry veterinarians are working to reduce the use of antimicrobials, as part of a conjoined response to the emerging global health threat of antimicrobial resistance.^16^ In 2018, this led to a reduction in use of 55% in broiler chickens since 2015, and a reduction in use of 13% in laying hens since 2016. A further 97% reduction has been recorded in the use of highest priority critically important antimicrobials (HPCIAs) in meat poultry (broiler and turkeys), compared to 2015.^17^ In 2018, the most commonly prescribed active ingredient in commercial meat poultry included penicillins (63% of total) and tetracyclines (16% of total).^17^ For commercial laying hens, tetracyclines (60% of total) and pleuromutilins (20% of total) were the most common.^17^ By recognising infections at an early stage, tailored treatment programmes can be applied. For backyard birds, it is unknown whether withdrawal periods for prescribed antimicrobials are being discussed with clients, as such periods can be for up to 21 days for egg laying hens. Prevention of viral infections is typically through biosecurity and vaccination in the commercial sector,^18^ however, backyard birds may have not received any vaccines. Ex‐farm birds might have received a vaccination earlier in their life, although these pre‐lay vaccinations are less likely to provide prolonged protection. Aspects of biosecurity are often implemented to only a limited extent in backyard chickens,^19^ leading to increased infection risk.

Globally, a number of publications have presented data from both an owner and animal perspective for backyard poultry.^20^, ^21^, ^22^, ^23^ Such studies have identified endemic infectious diseases such as *Mycoplasma gallisepticum, Mycoplasma synoviae, Salmonella enterica, Escherichia coli* and IBV in backyard poultry, which draws parallels with the commercial sector.^11^, ^24^, ^25^, ^26^ Previous work in the UK has been focused on a regional level,^13^, ^27^ and national‐level owner demographic features are yet to be determined. Data for the Greater London Urban Area showed that vaccination of backyard birds was rare, and that biosecurity measures were not strictly adhered to.^10^ A lack of knowledge of the legislation surrounding poultry ownership was also highlighted,^27^ which may contribute towards a lower number of voluntarily registered premises. Additionally, owners demonstrated limited knowledge of both avian‐restricted and zoonotic diseases. These factors are likely to lead to a reduction in bird welfare and hamper the ability to identify early onset of infection. Poultry, as prey species, also tend to present relatively subtle early clinical signs, further complicating matters. As such, monitoring is required to establish the prevalence of infectious pathogens in small and backyard poultry flocks, ideally through diagnostic investigations in the affected birds but also by assessing the prevalence of different clinical signs.

The Small Animal Veterinary Surveillance Network (SAVSNET) collects anonymised electronic health records (EHRs) from small animal practices in near real time.^28^ Such EHRs can record presenting clinical signs, differential diagnoses, treatment and outcomes. Limited anonymised demographic data can also be recorded. Hence this study aimed to summarise and characterise EHR data on backyard poultry that were collected by UK small animal practices between 2014 and 2019.

## MATERIALS AND METHODS

### Data collection and curation

SAVSNET collected EHRs in near real‐time from completed consultations in voluntary veterinary practices between 1st April 2014 and 31st March 2019. EHRs were read to confirm the presence of poultry. Poultry were defined as the following avian species as stated by DEFRA and the APHA: chickens, turkeys, ducks, geese, guinea fowl, quail, partridges, pheasants and pigeons.^2^ Once EHRs related to poultry were identified, the following demographic variables were extracted: species, breed, size of flock and ex‐farm status. To provide information about the location of where poultry likely resided, the owner postcode area was also extracted.

A list of clinical signs was created in consultation between all authors. Clinical narratives associated with each consultation were read by two authors (Cameron Rennie and John S.P. Tulloch) and then coded to note whether each clinical sign was recorded. Additionally, it was noted whether a conversation was held about drug withdrawal limits with the client, and whether the consultation ended in euthanasia. Pharmaceutical interventions (grouped into 14 pharmaceutical classes) were identified via reference to charged items associated with each consultation, as fully described previously.^29^ As time between performance and payment for euthanasia can be variable, a combination of clinical narrative identification and item charging was used to identify relevant consultations. All data collection has been ethically approved by the University of Liverpool's ethical review committee (reference: RETH000964).

### Demographic analysis

Data relating to species and demographic variables were described. Consultation rates were defined as the number of poultry consultations per total number of all animal consultations (including multiple consultations for the same animal), calculated at monthly and annual rates. To understand poultry geographic distribution, the number of unique animals regardless of species in the SAVSNET database, for the entire study period, was stratified by animal owners' postcode area. Using this, the incidence of poultry per SAVSNET population by postcode area was calculated, and a poultry distribution map created. Postcode area data were linked to Office of National Statistics demographic data to provide information about the area in which poultry resided.^30^ These geographically linked data are only available for England, and as such demographic analysis has been restricted to England rather than the UK. The two variables of interest were rural‐urban status and Index of Multiple Deprivation (IMD). The annual incidence of poultry residing in rural and urban areas was calculated; 95% confidence intervals (CIs) were calculated using Byar's method. An incidence rate ratio was calculated using a median‐unbiased estimation, with 95% CIs calculated by exact methods. IMD describes the relative deprivation of geographic small areas in England.^31^ The first decile contains the most deprived areas, while the tenth decile is the least deprived. Incidence per IMD decile was created, using Byar's method for 95% CIs. These were plotted and described.

### Clinical signs and treatment analysis

Recorded clinical signs and pharmaceutical interventions were described as a percentage of total chicken consultations, using proportions and 95% CIs taking account of clustering by veterinary practice (bootstrap method, n = 5000 samples). The most frequently prescribed pharmaceutical agents within each described class were also summarised, as was frequency of co‐presenting clinical signs and co‐prescription of pharmaceutical agents.

Clinical signs associated with an antimicrobial or anti‐inflammatory being prescribed, or euthanasia being performed, were also identified via three separate multivariable mixed effects logistic regression models. In each case, presence or absence of an antimicrobial, anti‐inflammatory or euthanasia being provided within a consultation was first univariably modelled as binary outcome variables against a range of clinical signs, modelled as binary explanatory variables. For antimicrobial and anti‐inflammatory models, presence of prescription of other pharmaceutical classes was also modelled. For all models, a likelihood ratio test (LRT) of null models indicated inclusion of practice, practice site and individual animal as random effects to provide best model fit. Univariable models were retained for multivariable analyses if an LRT indicated *p <* 0.20 against a null model.

Multivariable analysis underwent step‐wise backward elimination to reduce Akaike Information Criterion (AIC) and Bayesian Information Criterion (BIC). Confounding was accounted for via assessment of effect variation upon removal of variables. Two‐way interaction terms were assessed for improved multivariable model fit via a combination of AIC and BIC; for all three models no interaction terms were found to improve fit. Multicollinearity was assessed in the final models via use of the Variance Inflation Factor, available through the R package ‘car’. Statistical significance was defined as *p* < 0.05, and all analyses were carried out using R version 3.4.1 (R Core Team, 2017).

## RESULTS

### Demographic results

Between 1st April 2014 and 31st March 2019, 4424 unique poultry EHRs from 197 veterinary practices (352 sites) were summarised. Chickens made up 84.5% (n = 3,740) of consults, followed by ducks (9.6%, n = 424), pigeons (3.3%, n = 145), geese (1.8%, n = 79), quail (0.5%, n = 20), turkeys (0.2%, n = 10), pheasant (0.07%, n = 3), partridge (0.05%, n = 2) and guinea fowl (0.02%, n = 1). Due to the predominance of chickens, all remaining analyses focused solely on chickens.

Chicken breed was not recorded in 96.3% (n = 3,601) of consultations. In total, 55 breeds were recorded and only ‘bantam’ was recorded in more than 10 EHRs. Flock size was recorded in 12.0% (n = 449) of consultations. Of these, 85.3% (n = 383) mentioned flock sizes of less than 10 chickens, 9.8% (n = 44) had between 11 and 20 chickens, 2.4% (n = 11) had 21–30, 0.9% (n = 4) had 31–40 and 0.4% (n = 2) contained 40–49. One per cent of consults (n = 5) recorded 50 or more birds. Origin was recorded in 5% (5.3%, n= 197) of narratives, all being ex‐farm.

**FIGURE 1:**
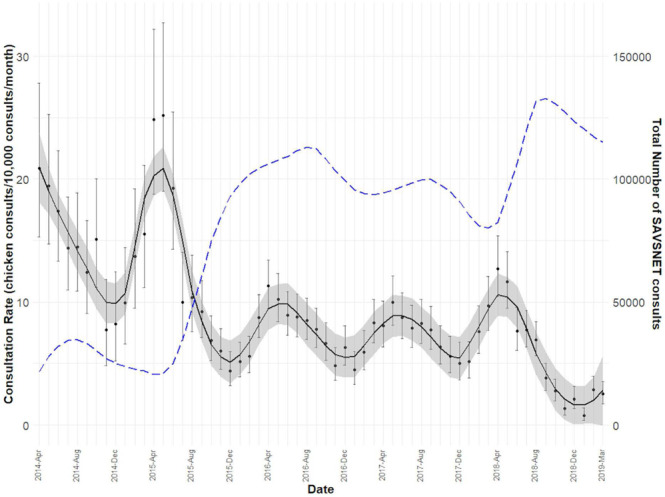
Monthly consultation rate of chickens presenting to veterinary primary care (black line) and monthly consultation rate of all SAVSNET consultation (blue dashed line). Smoothed trend lines created using a LOESS method

Mean annual consultation rate was 7.4 (95% confidence interval, CI 7.2–7.6) chicken consultations per 10,000 of all species consultations (Figure 1). In 2015 it was 9.9 (CI 9.1–10.8) chicken consultations per 10,000 consultations, declining to 7.8 (CI 7.3–8.3) in 2016, 7.2 (CI 6.8–7.7) in 2017 and 6.1 (CI 5.7–6.6) in 2018. Monthly consultation rate figures displayed clear annual seasonality, with peaks in early summer (April, May and June), and troughs in December.

**FIGURE 2:**
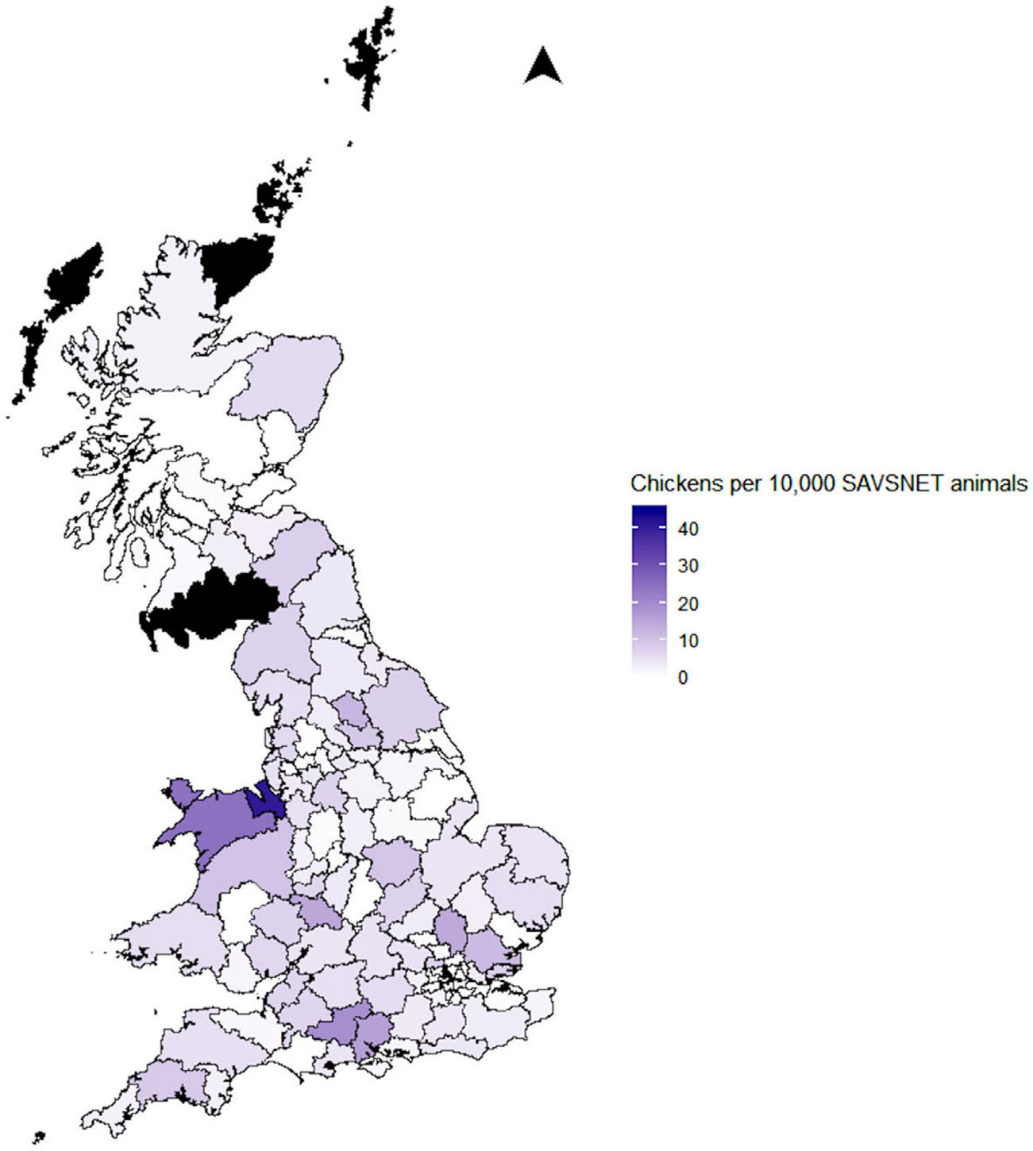
Mean annual incidence of chickens in veterinary primary care. (Black areas recorded no SAVSNET consultations)

No clear geographic trends were identified (Figure 2). Mean and median annual incidence of chickens per 10,000 animals by postcode area was 4.0 (CI 2.7–10.5) and 2.8 (range 0–41.1), respectively. Twenty per cent (20.2%, n = 14) of postcode areas recorded no chicken consultations during the study period. The top three postcode areas were: Chester (41.1, CI 28.6–57.2), Salisbury (19.1, CI 14.7–35.8) and Llandudno (16.1, CI 16.1–35.8).

**FIGURE 3:**
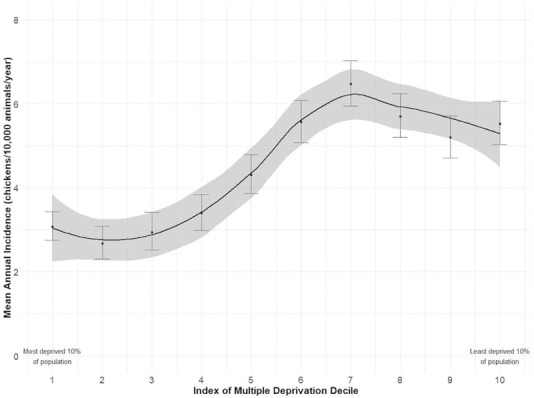
Mean annual incidence of chickens in veterinary primary care by level of societal deprivation. Smoothed trend line created using a LOESS method

Of chickens residing in England (n = 3,486), mean annual incidence of chickens residing in rural areas was significantly higher (7.9 chickens per 10,000 SAVSNET animals, CI 7.6–8.3) compared to those residing in urban area (3.2, CI 3.1–3.4, incidence rate ratio 2.5, CI 2.3–2.6, p < 0.001). The overall trend for IMD showed that as the levels of deprivation declined, the number of chickens increased (Figure 3). However, this relationship was not linear but sigmoid, and incidence peaked in the seventh decile; there were more chickens originating in the least deprived parts of England.

### Clinical signs

**FIGURE 4:**
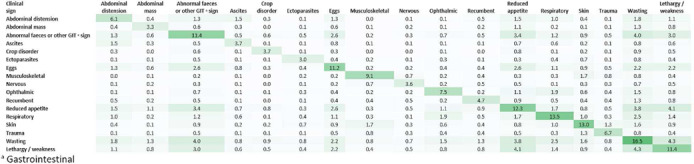
Co‐presenting clinical signs, summarising clinical signs recorded in 2% of more total chicken consultations, as a percentage of total recorded chicken consultations

Of total chicken consultations (n = 3,740), the most commonly recorded clinical sign was ‘wasting’, representing 16.6% of total consultations (CI 15.1–18.0), followed by respiratory clinical signs (13.6%, CI 12.1– 15.2) and reduced appetite (12.5%, CI 10.9–14.1). General health check‐ups were described in 16.2% of consultations (CI 14.7–17.8), although birds suffering non‐specific signs of ill health were the most frequently recorded consultation type (17.6%, CI 15.9–19.2). A blank or unclear narrative, not allowing adequate case summary, was recorded in 7.8% (CI 6.1–9.6) of consultations (Table 1). Of note, lethargy/weakness was commonly associated with wasting and reduced appetite, with such co‐presentations being recorded in 4.3% and 4.1% of total consultations, respectively (Figure 4).

**TABLE 1:** Percentage of backyard chicken consultations in which a set of clinical signs were recorded in the associated clinical narrative

Clinical sign	% of EHRs^a^ (CI^b^)
Unspecific unwell	17.6 (15.9–19.2)
Wasting	16.6 (15.1–18.0)
General health check	16.2 (14.7–17.8)
Respiratory	13.6 (12.1–15.2)
Skin	13.0 (11.7–14.3)
Reduced appetite	12.5 (10.9–14.1)
Lethargy/weakness	11.6 (10.0–13.3)
Abnormal faeces or other GIT^c^ sign	11.4 (10.3–12.6)
Eggs^d^	11.3 (9.6–13.0)
Musculoskeletal	9.0 (8.1–10.0)
Unknown/blank narrative	7.8 (6.1–9.6)
Ophthalmic	7.5 (6.5–8.5)
Other clinical sign(s)	7.2 (6.3–8.1)
Trauma	6.8 (5.9–7.6)
Abdominal distension	6.3 (4.8–7.7)
Recumbent	4.7 (3.9–5.5)
Crop disorder	3.9 (3.2–4.6)
Ascites	3.7 (2.8–4.7)
Nervous	3.5 (2.8–4.2)
Abdominal mass	3.3 (2.7–3.8)
Ectoparasites	3.0 (2.4–3.6)
Found dead^e^	2.1 (1.6–2.6)
Cyanosis	1.8 (1.4–2.2)
Anaemia	1.7 (1.3–2.1)
Postural	1.6 (1.1–2.0)
Swollen head	1.0 (0.6–1.4)
Torticollis	0.6 (0.4–0.9)
Oral canker	0.4 (0.2–0.6)
Behavioural	0.4 (0.2–0.6)
Endoparasites	0.3 (0.1–0.5)

a Electronic health record.

b 95% confidence interval.

c Gastrointestinal.

d ‘Eggs’ refer to reduction or cessation in egg laying or egg malformations.

e ‘Found dead’ refer both to chickens found dead at their home and those presented to the practice dead.

### Pharmaceutical prescription

Antimicrobial agents were the most commonly prescribed pharmaceutical class, having been prescribed in 33.0% (CI 29.8–36.2) of total chicken consultations (Table 2). Both an antimicrobial and anti‐inflammatory of any type were prescribed in 9.1% of total consultations. Considering individual pharmaceutical agents, preference for a particular or small number of agents within a class was apparent across most classes. A conversation about drug withdrawal limits was recorded as having occurred in 11.8% (CI 10.8–12.9) of consultations. Euthanasia was described in the clinical narrative or charged for in 29.8% of total consultations (CI 27.0–32.6).

**TABLE 2:** Pharmaceutical prescription by class and most commonly prescribed agent, as a percentage of total consultations and as a percentage of total prescription events within the described class

Pharmaceutical class	% EHRs^a^ (CI^b^)	Top agent	% EHRs	% class prescriptions
Anti‐inflammatory	16.4 (13.5–19.3)	Meloxicam	14.3	88.2
Antimicrobial	33.0 (29.8–36.2)	Enrofloxacin	13.2	40.6
Antimycotic	0.3 (0.1–0.5)	Nystatin	0.1	46.7
Cardiovascular	0.6 (0.2–0.9)	Frusemide	0.5	83.3
Ectoparasiticide	0.4 (0.1–0.6)	Fipronil	0.4	90.0
Endectocide	2.6 (1.8–3.4)	Ivermectin	2.7	100.0
Endoparasiticide	2.4 (1.7–3.1)	Fenbendazole	0.9	37.5
Gastrointestinal	0.6 (0.1–1.1)	Metoclopramide	0.5	83.3
Hormone	1.4 (0.7–2.1)	Deslorelin	1.2	85.7
Neurological^c^	0.6 (0.4–0.9)	Non‐specified	0.3	33.3
Ocular	0.1 (0.0–0.2)	Fluoroscein	0.1	100.0
Replacement agent	1.6 (1.0–2.2)	Calcium gluconate	0.4	25.0
Respiratory^d^	0.0 (0.0–0.1)	Bromhexine	0.0	100.0
Euthanasia dispensed	27.0 (24.4–29.6)	–	–	–
Euthanasia ‐ narrative	22.7 (20.2–25.2)	–	–	–
COMBINED euthanasia	29.8 (27.0–32.6)	–	–	–

a Electronic health record.

b 95% confidence interval.

c Most common specified agent: Butorphanol (0.2% EHRs).

d One prescription event.

Although enrofloxacin was the most commonly prescribed antimicrobial agent (40.6% of total antimicrobial prescription events), other agents were relatively commonly prescribed too, including tylosin (19.5% total antimicrobial prescription events), clavulanic acid potentiated amoxicillin (12.1%) and oxytetracycline (6.5%). HPCIAs were prescribed in 43.8% of total antimicrobial prescribing events in 14.3% of total consultations; such prescriptions almost entirely consisted of fluoroquinolones. In total, 92.4% of antimicrobial prescribing consultations included formulations authorised for systemic (oral or injectable) administration alone; 5.2% topical administration alone, and 1.8% consultations were associated with both systemic and topical antimicrobial agent prescription.

### Clinical signs and pharmaceutical prescription

#### Antimicrobial prescription

A range of clinical signs were associated with significantly increased odds of antimicrobial prescription, most notably respiratory clinical signs (odds ratio [OR] 4.78, CI 3.69–6.19), as well as signs associated with dermatological, or ophthalmic disease. Prescription of anti‐inflammatories (OR 5.29, CI 4.11–6.83) or neurological agents (OR 4.69, CI 1.69–13.05) within the same consultation were also associated with increased odds of antimicrobial prescription. Conversely, musculoskeletal (OR 0.40, CI 0.28–0.56) or nervous (OR 0.57, CI 0.34–0.96) disease, in addition to signs potentially indicative of advanced disease (i.e. recumbency, OR 0.20, CI 0.12–0.35), was associated with reduced prescription odds (Table 3).

**TABLE 3:** Multivariable mixed effects logistic model findings exploring odds of antimicrobial prescription against a range of clinical signs and presence of prescription of other pharmaceutical classes. Individual practice variance was 0.27 (standard deviation [SD] 0.52), individual site variance was 0.14 (SD 0.37) and individual animal variance was 0.39 (SD 0.62). Univariable analyses are summarised in Supplementary material, Table 1

Variable	Beta	SE^a^	OR^b^ (CI^c^)	*p* value
Intercept	‐1.50	0.10	–	–
		**Clinical signs**		
**Abdominal mass**	**−0.90**	**0.28**	**0.41 (0.24–0.70)**	**0.001**
**Abnormal faeces or other GIT**	**0.78**	**0.14**	**2.18 (1.67–2.85)**	<**0.001**
Behavioural	‐1.79	1.12	0.17 (0.02–1.50)	0.110
**Crop disorder**	**−0.89**	**0.26**	**0.41 (0.25–0.69)**	**0.001**
**Musculoskeletal**	**−0.93**	**0.17**	**0.40 (0.28–0.56)**	<**0.001**
**Nervous**	**−0.57**	**0.27**	**0.57 (0.34–0.96)**	**0.035**
**Ophthalmic**	**1.27**	**0.16**	**3.55 (2.58–4.89)**	<**0.001**
**Recumbent**	**−1.59**	**0.28**	**0.20 (0.12–0.35)**	<**0.001**
**Respiratory**	**1.56**	**0.13**	**4.78 (3.69–6.19)**	<**0.001**
**Skin**	**0.50**	**0.13**	**1.64 (1.28–2.11)**	<**0.001**
**Swollen head**	**1.02**	**0.46**	**2.76 (1.11–6.85)**	**0.028**
**Unwell**	**0.24**	**0.12**	**1.27 (1.01–1.59)**	**0.037**
**Lethargy/weakness**	**0.29**	**0.14**	**1.34 (1.02–1.75)**	**0.034**
		**Pharmaceutical prescription**		
**Anti‐inflammatory**	**1.67**	**0.13**	**5.29 (4.11–6.83)**	<**0.001**
**Neurological**	**1.55**	**0.52**	**4.69 (1.69–13.05)**	**0.003**

a Standard error.

b Odds ratio.

c 95% confidence interval.

#### Anti‐inflammatory prescription

A range of clinical signs were associated with significantly increased odds of anti‐inflammatory prescription, most notably musculoskeletal clinical signs (OR 7.38, CI 5.20–10.49), trauma, postural and dermatological disease. Prescription of antimicrobials (OR 4.93, CI 3.82–6.37), antimycotics (OR 6.07, CI 1.27–28.89) or neurological agents (OR 4.65, CI 1.65–13.12) within the same consultation were also associated with increased odds of anti‐inflammatory prescription. Conversely, check‐ups were associated with decreased odds (OR 0.67, CI 0.49–0.92), as were wasting and gastroenteric clinical signs (Table 4).

**TABLE 4:** Multivariable mixed effects logistic model findings exploring odds of anti‐inflammatory prescription against a range of clinical signs and presence of prescription of other pharmaceutical classes. Individual practice variance was <0.01 (SD < 0.01), individual site variance was 0.38 (SD 0.62), and individual animal variance was 0.55 (SD 0.74). Univariable analyses are summarised in Supplementary material, Table 2

Variable	Beta	SE^a^	OR^b^ (CI^c^)	*p* value
Intercept	‐3.02	0.18	**–**	***–***
		**Clinical signs**		
Unknown/blank narrative	‐0.49	0.26	0.62 (0.37–1.03)	0.065
**Abnormal faeces or other GIT**	**−0.39**	**0.19**	**0.68 (0.47–0.98)**	**0.039**
**Check‐up**	**−0.40**	**0.16**	**0.67 (0.49–0.92)**	**0.014**
**Musculoskeletal**	**2.00**	**0.18**	**7.38 (5.20–10.49)**	<**0.001**
**Postural**	**1.60**	**0.35**	**4.96 (2.47–9.94)**	<**0.001**
**Skin**	**0.49**	**0.15**	**1.62 (1.21–2.19)**	**0.001**
**Swollen head**	**1.61**	**0.43**	**5.03 (2.19–11.57)**	<**0.001**
**Trauma**	**1.03**	**0.19**	**2.79 (1.91–4.08)**	<**0.001**
**Wasting**	**−0.40**	**0.16**	**0.67 (0.49–0.92)**	**0.014**
		**Pharmaceutical prescription**		
**Antimicrobial**	**1.60**	**0.13**	**4.93 (3.82–6.37)**	<**0.001**
**Antimycotic**	**1.80**	**0.80**	**6.07 (1.27–28.89)**	**0.024**
Endectocide	‐0.77	0.41	0.46 (0.21–1.03)	0.059
**Neurological**	**1.54**	**0.53**	**4.65 (1.65–13.12)**	**0.004**
**Replacement agent**	**1.05**	**0.37**	**2.87 (1.38–5.96)**	**0.005**

a Standard error.

b Odds ratio.

c 95% confidence interval.

#### Euthanasia

A range of clinical signs were significantly associated with increased odds of euthanasia, most notably recumbency (OR 7.22, CI 4.85–10.75), an abdominal mass, neurological clinical signs and wasting. Conversely, check‐ups were associated with decreased odds (OR 0.26, CI 0.19–0.34), as were a range of dermatological, musculoskeletal or ophthalmic clinical signs (Table 5).

**TABLE 5:** Multivariable mixed effects logistic model findings exploring odds of euthanasia against a range of clinical signs. Individual practice variance was 0.12 (SD 0.35), individual site variance was 0.18 (SD 0.42), and individual animal variance was 0.04 (SD 0.21). Univariable analyses are summarised in Supplementary material, Table 3

Variable	Beta	SE^a^	OR^b^ (CI^c^)	*p* value
Intercept	‐0.74	0.09	–	
Unknown/blank narrative	1.02	0.16	2.77 (2.05–3.76)	<0.001
Abdominal distension	0.44	0.17	1.56 (1.13–2.16)	0.007
Abdominal mass	1.03	0.21	2.80 (1.85–4.24)	<0.001
Abnormal faeces or other GIT	‐0.65	0.14	0.52 (0.39–0.69)	<0.001
Check‐up	‐1.36	0.15	0.26 (0.19–0.34)	<0.001
Eggs	‐0.64	0.14	0.53 (0.40–0.69)	<0.001
Musculoskeletal	‐0.58	0.16	0.56 (0.41–0.77)	0.002
Nervous	1.00	0.21	2.71 (1.80–4.08)	<0.001
Ophthalmic	‐0.75	0.18	0.47 (0.33–0.68)	<0.001
Oral canker	‐2.09	1.06	0.12 (0.02–0.98)	0.048
Other	0.55	0.15	1.74 (1.30–2.33)	<0.001
Recumbent	1.98	0.20	7.22 (4.85–10.75)	<0.001
Respiratory	‐0.74	0.14	0.48 (0.36–0.63)	<0.001
Skin	‐1.19	0.16	0.30 (0.22–0.41)	<0.001
Swollen head	‐1.31	0.63	0.27 (0.08–0.94)	0.039
Wasting	0.51	0.11	1.67 (1.34–2.08)	<0.001
Lethargy or weakness	0.25	0.13	1.29 (1.00–1.65)	0.051

a Standard error.

b Odds ratio.

c 95% confidence interval.

## DISCUSSION

This is the first nationwide study to describe the UK backyard chicken flock and their presenting health conditions to small animal veterinarians. It has highlighted a number of concerns relating to welfare, antimicrobial resistance and public health. Birds presenting with advanced stages of disease or unspecific clinical signs were common, indicating a potential lack of owner knowledge for recognising early‐stage clinical signs, and potentially limited practitioner ability to fully clinically assess a chicken. These factors combined might well explain the high level of euthanasia (29.9% of all chicken consults) observed in this study. Hence, this study has identified several opportunities to improve both practitioner and owner knowledge of potential health conditions in backyard poultry. These interventions would lead to improved bird welfare.

Unsurprisingly, chickens were the predominant poultry type to present to small animal veterinarians, making up 84.5% of consults. Only 5.3% of consults reported the origin of the bird, all of which were ex‐farm. This is potentially a large underestimate as 200,000 hens were rehomed in the UK between 2005 and 2012.^13^ Understanding bird type and origin is important as it may impact differential diagnosis, based on potential vaccination and exposure risks of such birds.

Poultry consultations seemingly declined over the time period investigated, which occurred alongside a reported slight decline in the UK domestic fowl pet population (0.7 million in 2014–15 to 0.5 million in 2018–19).^1^ Although it should be noted that observed variations here could be due to varied and expanding practice participation, perhaps producing a bias towards more urban practices less likely to see poultry over this time period, seasonality in relative poultry consultation frequency was consistently noted. Peaks in early summer were observed; a period typically associated with a reduction in precipitation and an increase in air temperature in the UK.^32^ Commercial poultry owners monitor and control environmental conditions, including temperature, humidity and lighting.^33^, ^34^ However, maintaining optimal conditions over the full year has difficulties for backyard owners. Despite the UK climate being temperate, heat stress and humidity can still impact laying hen's health, by exacerbating potential infections or reducing laying rates,^35^, ^36^ a finding perhaps evidenced in the seasonality in poultry presentation frequency noted in the current study. It is also possible that improved weather associated with summer months might enhance contact frequency between owners and their birds, increasing probability of noticing ill health.

There was relatively low scale chicken ownership across the UK, with no obvious parts of the country with increased or decreased ownership. SAVSNET pet owners were 2.5 times more likely to own chickens if they lived in rural areas. This supports survey data from Scotland where 63.1% of backyard poultry were kept in the countryside and 22.7% in rural villages.^27^ Backyard poultry presentation to veterinary practices is likely to predominate in practices serving rural and semi‐rural communities. In this study, we found that the 40% least deprived neighbourhoods had the highest chicken ownership. It is important to remember that deprivation is not a reflection of class or wealth status, but a measure of relative deprivation. It is measured for a neighbourhood rather than a household, and so ecological fallacy must be acknowledged. The reason for those residing in areas with more societal and material benefits being more likely to visit a veterinarian with their chickens is unknown. Results may be biased in that those in more deprived areas may not have the means to take their chicken to a vet, or they may value their chicken differently. Further research is needed to explore the demographics and views of UK backyard poultry owners.

The most prominent clinical sign documented in EHRs was non‐specific illness, which indicates a potential knowledge chasm when compared with dogs, cats and rabbits.^37^ It also highlights a potential opportunity for small animal veterinarians to further their knowledge of poultry, especially as the third most common sign reported on the APHA Avian Disease Surveillance Dashboard^12^ is also ‘other/unwell’. The highest reported grouped clinical sign was ‘respiratory’, commonly associated with infectious disease, further suggesting that backyard poultry can provide a reservoir for respiratory pathogens. This can facilitate spread of disease to other flocks through sales, markets or shows.^38^ Preventative health was of a lower prevalence when compared to other animal species kept as pets^29^; however, the higher rate of euthanasia reported in backyard chickens would have impacted this ratio.

An important finding throughout this study was the high prescription rate of HPCIAs, such as enrofloxacin. This is of particular concern, as efforts by the commercial sector have seen a 97% reduction in HPCIA use.^17^ However, while the reason behind their prescription is not known, it may be due to limited access to those used in the commercial sector,^39^ or a lack of confidence to prescribe some antimicrobials. Limited antimicrobial products are authorised for use in poultry, especially for non‐food producing birds. While enrofloxacin is authorised, it is not for use where eggs are produced for human consumption. Further, we identified prescription of fipronil, an ectoparasiticide not authorised for animal and animal products intended for human consumption, including poultry. Owners should be made aware that eggs produced by enrofloxacin or fipronil‐treated birds must never be used for human consumption. As most owners keep backyard chickens for eggs, the importance of discussing withdrawal periods with owners therefore cannot be stressed enough.^40^ In the current study, despite antimicrobials or anti‐inflammatories being prescribed in 32.5% and 16.1% of EHRs respectively, only 11.8% recorded that a withdrawal conversation had taken place. It should be noted that this latter figure represents a minimum value for withdrawal conversations, as the topic might have been discussed during the consultation but not recorded in the clinical narrative. Increased education of suitable pharmaceutical agents, and their relevant withdrawal periods, including the importance of recording such conversations in the clinical narrative, would nevertheless be of benefit for poultry, owners and the general public alike.

Of interest, this study further identified several clinical signs associated with increased odds of antimicrobial prescription, such as ‘respiratory’ and ‘ophthalmic’. Such associations have previously been identified in other species and are likely to reflect a perception that these disease manifestations are most commonly bacterially mediated. Although bacterial respiratory pathogens (e.g. *M.gallisepticum, M.synoviae*) are considered to be widespread in small poultry flocks,^41^ respiratory disease can also be viral or non‐infectious in origin.^42^ Anti‐inflammatories were frequently co‐prescribed with antimicrobials. In dogs and cats, anti‐inflammatory prescription frequency during respiratory disease consultations has been increasing in recent years, coupled with a comparative reduction in antimicrobial prescription frequency.^43^ This suggests increased broader veterinarian recognition of the non‐infectious component to respiratory disease. We hypothesise whether such changing attitudes could be further utilised to prompt comparative improvements in veterinarian approach to antimicrobial prescription in backyard poultry.

Euthanasia rates were relatively higher (29.9% of EHRs) when compared to those of companion animals, such as dogs and cats,^29^ and were frequently associated with clinical signs more commonly attributed to increased disease severity (e.g. recumbency). While the reasons contributing to this are not explicitly known, previous work has suggested limited owner awareness of health issues in poultry.^13^ An increased euthanasia rate may also suggest how owners interpret and identify signs, particularly as signs such as emaciation may only be observed through regular handling or observation by owners. Although it is not surprising that increased disease severity would be associated with increased risk of euthanasia, these findings suggest that owners might only identify illness at a late stage, where the ability to positively impact on disease outcome through veterinary intervention is limited. The relative value of a chicken compared to costs of treatment is also a consideration for owners^44^ and may delay a decision to present a backyard chicken to a veterinarian until the disease course is too advanced for a positive outcome to be realistic or affordable. A 2014 UK survey reported that only 17% of responding veterinarians dealt with both small animal and poultry practice, compared with 58% who were strictly small animal only.^45^ With backyard poultry owners primarily using small animal practices, most practices will likely not have a poultry specialist, and clinical knowledge of poultry may be limited. More work is clearly needed to understand the value owners and veterinarians place on backyard poultry, and how this might influence veterinary care and welfare. Nevertheless, there is significant scope for focused education to improve backyard poultry veterinary interactions.

There are a number of limitations that should be considered assessing the merits of this study. SAVSNET practices are selected by convenience, and hence might not be representative of all UK veterinary practices. SAVSNET focuses on booked consultations with a veterinary professional; hence, interactions and product sales outside of the consultation environment would be missed. Although significant efforts were made to identify animal species, demographic features, clinical signs and pharmaceutical prescriptions through manual and semi‐automated text mining methodologies, respectively, it is possible that descriptions lacking clarity or absent from the EHR might have been missed by the authors. It should be remembered that risk factor modelling only identifies associations between the outcome variable of interest and explanatory variables and does not attribute causation.

## CONCLUSION

Our results highlight that there may be limited knowledge and engagement of both chicken owners and primary care veterinarians regarding backyard poultry, as evidenced by relatively advanced disease presentations and high euthanasia rates. With the continued popularity of backyard poultry, now is the perfect time for vets to upskill and become more aware of poultry management and health problems. By doing so we will do much to improve the health, welfare and husbandry of the UK backyard poultry flock.

## ACKNOWLEDGEMENTS

This work was generously funded by the Animal and Plant Health Agency and further benefited from support provided by the INSPIRE Summer Research School programme (www.liverpool.ac.uk/veterinary‐science/research/inspire/summer‐research‐school/). SAVSNET is grateful for the support and major funding from the BBSRC and BSAVA. We wish to thank data providers in veterinary practice (VetSolutions, Teleos, CVS and independent practitioners) and participating veterinary diagnostic laboratories (Axiom Veterinary Laboratories, Batt Laboratories, BioBest, Idexx, NationWide Laboratories Microbiology Diagnostics Laboratory at the University of Liverpool, the Department of Pathology and Infectious Diseases at the University of Surrey, and the Veterinary Pathology Group), without whose support and participation this research would not be possible. Finally, we are especially grateful for the help and support provided by SAVSNET team members Bethaney Brant, Susan Bolan and Steven Smyth.

## FUNDING INFORMATION AND CONFLICT OF INTEREST

Throughout this work, David A. Singleton was funded by BBSRC. Cameron Rennie, David A. Singleton and John S.P. Tulloch were funded by INSPIRE (The Academy of Medical Sciences). The authors declare no conflict of interest.

## AUTHOR CONTRIBUTIONS

John S.P. Tulloch and David A. Singleton conceived the project idea, and it was refined through collaboration with Phil H. Jones and David Welchman. Christopher Ball, David A. Singleton and John S.P. Tulloch designed the methodology with the assistance of David Welchman, Phil H. Jones, Kannan Ganapathy. Cameron Rennie and Charlotte Coxon performed initial data extraction and preliminary analysis. David A. Singleton and John S.P. Tulloch performed further analysis. Christopher Ball, David A. Singleton and John S.P. Tulloch wrote the paper manuscript in consultation with Phil H. Jones, David Welchman and Kannan Ganapathy.

## SUPPORTING INFORMATION

Additional supporting information may be found online in the Supporting Information section at the end of the article.

**How to cite this article**: Singleton DA, Ball C, Rennie C, et al. Backyard poultry cases in UK small animal practices: Demographics, health conditions and pharmaceutical prescriptions. *Vet Rec*. 2021;e71. https://doi.org/10.1002/vetr.71
